# Animal Models of Diabetes and Metabolic Disease 2014

**DOI:** 10.1155/2015/571809

**Published:** 2015-04-20

**Authors:** Tomohiko Sasase, Norihide Yokoi, Marcus G. Pezzolesi, Masami Shinohara

**Affiliations:** ^1^Biological/Pharmacological Research Laboratories, Central Pharmaceutical Research Institute, Japan Tobacco Inc., Osaka 569-1125, Japan; ^2^Division of Molecular and Metabolic Medicine, Department of Physiology and Cell Biology, Kobe University Graduate School of Medicine, Kobe 650-0017, Japan; ^3^Section on Genetics and Epidemiology, Joslin Diabetes Center, Harvard Medical School, Boston, MA 02215, USA; ^4^Tokyo Animal & Diet Department, CLEA Japan, Inc., Tokyo 153-8533, Japan

Metabolic diseases include various disorders of metabolism. Diabetes is one of the major types of metabolic diseases and the number of diabetic patients is still increasing worldwide. Complications associated with diabetes, such as retinopathy, peripheral neuropathy, and nephropathy, threaten quality of life (QOL). Diabetes is frequently associated with dyslipidemia, a risk factor for premature atherosclerosis and cardiovascular disease. Hypertension also causes cardiovascular diseases and stroke, and obesity is a known major risk factor for diabetes, cardiovascular diseases, and cancer. Furthermore, these diseases often coincide and closely affect each other.

To study these diseases, numerous hereditary animal models have been developed. Although molecular biological techniques have dramatically improved and have become more important to clarify underlying mechanisms of diseases, the importance of hereditary animal models has not changed. In this special issue we introduce recent beneficial experimental animal models in this field and present up-to-date information on the pathophysiology of and therapeutic drugs for metabolic diseases using valuable animal models. We hope that the 12 articles included in this special issue provide valuable information.

Similar to the previous special issue “Animal Models of Diabetes and Metabolic Disease” published in 2013, several new animal models will be introduced in this special issue. Four papers will present new diabetic animal models, the Spontaneously Diabetic Torii (SDT) rat and its derivative, the SDT fatty rat. The SDT rat, a nonobese type 2 diabetes (T2D) model, exhibits severe hyperglycemia associated with hypoinsulinemia and severe diabetic microvascular complications. F. Toyoda et al. evaluated the effect of administration of ranirestat, a novel aldose reductase inhibitor currently in clinical trials, on diabetic retinopathy and diabetic peripheral neuropathy and reported their findings in “Effect of Ranirestat, a New Aldose Reductase Inhibitor, on Diabetic Retinopathy in SDT Rats.” The SDT fatty rat is an obese T2D model established by introducing the* fa *allele in Zucker fatty rats into the SDT rat genome. Several articles introducing this new animal model were presented in the previous special issue. The discoverers of this model contribute additional information in this special issue. T. Ohta et al. mention gender differences in SDT fatty rats in their review article “Gender Differences in Metabolic Disorders and Related Diseases in Spontaneously Diabetic Torii-*Lepr* ^*fa*^ Rats.” Pathological perceptions regarding ocular inflammation in SDT fatty rats are reported in “Ocular Inflammation in Uveal Tract in Aged Obese Type 2 Diabetic Rats (Spontaneously Diabetic Torii Fatty Rats)” by Y. Kemmochi et al. The authors point out that this animal model has the potential for spontaneous uveitis. In the article entitled “Effects of Unilateral Nephrectomy on Renal Function in Male Spontaneously Diabetic Torii Fatty Rats: A Novel Obese Type 2 Diabetic Model,” Y. Katsuda et al. report findings from their investigation of the effect of nephrectomy on renal function and morphology in SDT fatty rats. These papers clearly show that the SDT fatty rat is useful in investigations to elucidate the pathogenesis of human diabetic microvascular complications.

In “Characterization of the Prediabetic State in a Novel Rat Model of Type 2 Diabetes, the ZFDM Rat,” G. Gheni et al. investigate the phenotypic characterization of a new obese T2D model, the Zucker fatty diabetes mellitus (ZFDM) rat. The authors also characterize insulin secretory responses to both glucose and GLP-1 stimulation in the isolated pancreatic islets. In addition to severe insulin resistance and diminished insulin response to incretin, the fragility of islets is related to the development of T2D in this animal model.

Animal models that show obesity, metabolic syndromes, and diabetes (e.g., the ZF rat, ZDF rat, SDT fatty rat,* ob/ob* mice, and* db/db* mice) exhibit mutations in the leptin pathway. However, leptin pathway mutations are uncommon in the human population. The ZDSD/Pco (ZDSD) rat exhibits polygenic obesity and diabetes without defects observed in the leptin pathway. R. G. Peterson et al. report the characteristics of the ZDSD rat in “Characterization of the ZDSD Rat: A Translational Model for the Study of Metabolic Syndrome and Type 2 Diabetes.” This new animal model may become a novel translational animal model for the study of human metabolic diseases and T2D.

Two pharmacological compounds effective against T2D and obesity were evaluated using diet-induced obesity (DIO)/diabetes or hereditary diabetic models in three articles. JTT-130 is a new intestine-specific microsomal triglyceride transfer protein (MTP) inhibitor expected to become a treatment for dyslipidemia, obesity, and diabetes. In the article “JTT-130, a Novel Intestine-Specific Inhibitor of Microsomal Triglyceride Transfer Protein, Reduces Food Preference for Fat,” Y. Mera et al. showed that JTT-130 specifically decreases total caloric intake by reducing the preference for fat and reducing body weight. S. Sakata et al. administered JTT-130 and pioglitazone to ZDF rats to investigate the effects of these drugs on glucose and lipid metabolism in the article “Combination Therapy of an Intestine-Specific Inhibitor of Microsomal Triglyceride Transfer Protein and Peroxisome Proliferator-Activated Receptor *γ* Agonist in Diabetic Rat.” Combination treatment with JTT-130 and pioglitazone resulted in intense glycemic control, strong hypolipidemic action, and an improvement in insulin sensitivity. These two articles suggest the possibility of using MTP inhibitors for the treatment of T2D associated with obesity and insulin resistance. JTT-551 is a new protein tyrosine phosphatase 1B (PTP1B) inhibitor and is a negative regulator of leptin signaling as well as insulin signaling that improves glucose metabolism by enhancing insulin signaling. In the article “Pharmacological Effects of JTT-551, a Novel Protein Tyrosine Phosphatase 1B Inhibitor, in Diet-Induced Obesity Mice,” M. Ito et al. showed that the antiobesity effects of JTT-551 may be due to the enhancement of leptin signaling and that the compound may be useful in the treatment of T2D and obesity.

Gestational diabetes and macrosomia that cause adulthood obesity are associated with several metabolic disorders. Beneficial effects of *ω*-3 fatty acids on cardiovascular diseases, as well as diabetes and obesity, have been proposed. In the review article “Beneficial Effects of Omega-3 Polyunsaturated Fatty Acids in Gestational Diabetes: Consequences in Macrosomia and Adulthood Obesity,” A. Yessoufou et al. summarized the effects of *ω*-3 PUFA, such as lowering high rates of macrosomia induced by diabetic pregnancy, reducing blood triglyceride levels, and protecting against oxidative stress. Based on these pharmacological effects, *ω*-3 PUFA is expected to become a treatment for gestational diabetes and inflammatory and immune diseases.

C1q/TNF-related protein-3 (CTRP3) is an adipokine that suppresses hepatic gluconeogenesis, thereby lowering blood glucose levels. X. Li et al. used high-fat diets plus a low-dose STZ-induced T2D model to investigate the expression of CTRP3 in a T2D model and to investigate the effects of GLP-1 receptor agonists in “Expression of CTRP3, a Novel Adipokine, in Rats at Different Pathogenic Stages of Type 2 Diabetes Mellitus and the Impacts of GLP-1 Receptor Agonist on It.” As highlighted in this article, decreased CTRP3 levels in diabetic visceral adipose tissue and insulin sensitivity improved with GLP-1 receptor agonist exendin-4 administration.

Roux-en-Y gastric bypass (RYGB) is a common bariatric operation to reduce body weight and treat T2D in obese patients. To evaluate the potential mechanisms of bariatric surgery, a large animal model with anatomical similarities that mimics human metabolic diseases is helpful. In the article “Evaluating the Mechanisms of Improved Glucose Homeostasis after Bariatric Surgery in Ossabaw Miniature Swine” by J. G. Sham et al., Ossabaw miniature swine are introduced as an animal model that mimics human metabolic syndromes to elucidate RYGB's influence on glucose homeostasis.

*Tomohiko Sasase* *Tomohiko Sasase* *Norihide Yokoi* *Norihide Yokoi* *Marcus G. Pezzolesi* *Marcus G. Pezzolesi* *Masami Shinohara* *Masami Shinohara*

